# Research Progress on Coating of Sensitive Materials for Micro-Hotplate Gas Sensor

**DOI:** 10.3390/mi13030491

**Published:** 2022-03-21

**Authors:** Zhenyu Yuan, Fan Yang, Fanli Meng

**Affiliations:** 1 College of Information Science and Engineering, Northeastern University, Shenyang 110819, China; yuanzhenyu@ise.neu.edu.cn (Z.Y.); yangfan13845850682@163.com (F.Y.); 2 State Key Laboratory of Synthetical Automation for Process Industries, Northeastern University, Shenyang 110819, China; 3 Hebei Key Laboratory of Micro-Nano Precision Optical Sensing and Measurement Technology, Qinhuangdao 066004, China; 4 Key Laboratory of Data Analytics and Optimization for Smart Industry, Ministry of Education, Northeastern University, Shenyang 110819, China

**Keywords:** MEMS, micro-hotplate gas sensor, sensitive material coating

## Abstract

Micro-hotplate gas sensors are widely used in air quality monitoring, identification of hazardous chemicals, human health monitoring, and other fields due to their advantages of small size, low power consumption, excellent consistency, and fast response speed. The micro-hotplate gas sensor comprises a micro-hotplate and a gas-sensitive material layer. The micro-hotplate is responsible for providing temperature conditions for the sensor to work. The gas-sensitive material layer is responsible for the redox reaction with the gas molecules to be measured, causing the resistance value to change. The gas-sensitive material film with high stability, fantastic adhesion, and amazing uniformity is prepared on the surface of the micro-hotplate to realize the reliable assembly of the gas-sensitive material and the micro-hotplate, which can improve the response speed, response value, and selectivity. This paper first introduces the classification and structural characteristics of micro-hotplates. Then the assembly process and characteristics of various gas-sensing materials and micro-hotplates are summarized. Finally, the assembly method of the gas-sensing material and the micro-hotplate prospects.

## 1. Introduction

Gas sensors play an essential role in modern life, and the demand for gas sensors is increasing day by day [1]. Traditional gas detection relies on chromatography spectroscopy [2]. The instruments used in these methods are large in size, high in price, and complex in operation, limiting field detection application [3]. Micro-nano gas sensors have attracted attention due to their small size [4], low price [5], and easy use. Micro-nano gas sensors are widely used in hazardous chemical detection [6], air quality monitoring [7], human health monitoring [8], chemical production process [9], intelligent systems for agricultural applications [10].

Among micro-nano sensors, MEMS micro-hot plate sensors are widely used due to their low power consumption [11], small size, and superior consistency [12]. The micro-hotplate gas sensor consists of a micro-hotplate and a gas-sensitive material layer. The micro-hotplate is responsible for providing temperature conditions for the sensor to work. The gas-sensing material layer is responsible for redox reactions with the gas molecules to be measured, causing changes in the resistance value and realizing the detection of gas types and concentrations. At present, there are many research papers on the synthesis of gas-sensing materials, but few reviews about the film-forming coating of gas-sensing materials are involved. The effective formation of patterned nano-scale gas-sensing material layers at specific locations on the micro-hotplate sensor surface is a major technical obstacle to be overcome in the fabrication of micro-hotplate gas sensors. The material film-forming technology on the surface of the micro-hotplate gas sensor is the key technology to realize the mass production and application of the micro-hotplate. The gas-sensitive material film with high stability, fantastic adhesion, and amazing uniformity is prepared on the surface of the micro-hotplate to realize the reliable assembly of the gas-sensitive material and the micro-hotplate, which can improve the response speed, response value, and selectivity.

This paper first introduces the classification and structural characteristics of micro-hotplates. Then the assembly process and characteristics of various gas-sensing materials and micro-hotplates are summarized. Finally, the future development of coating methods for gas-sensing materials is prospected.

## 2. Gas Sensitive Material Coating

The efficient formation of patterned sensor nanomaterials at specific positions on the surface of the micro-hotplate gas sensor and the rational integration of the patterning method with MEMS technology are the leading technical obstacles to be overcome in the fabrication of the micro-hotplate gas sensor [13]. The first part of this section introduces the classification and structural characteristics of micro-hotplates, and the second part introduces the assembly methods of different gas-sensitive materials and micro-hotplates, such as drop coating [14], liquid deposition [15], atomic layer deposition [16], chemical vapor deposition [17], dielectrophoretic deposition [18], spray pyrolysis [19], sputtering [20], in situ growth [21], inkjet printing [22], screen printing [23], self-assembly [24].

### 2.1. Micro-Hotplate Classification and Structure

Micro-nano fabrication technology promotes the development of sensor miniaturization. Ceramic tube gas sensor is made by coating-sensitive material on the outside of the ceramic tube, which has some shortcomings in the production process, such as low efficiency and consistency cannot be guaranteed. To overcome the shortcomings of ceramic tube gas sensors, researchers apply microelectronic manufacturing technology to the sensor-manufacturing process and design a micro-hotplate gas sensor. The micro-hotplate gas sensor has the advantages of low power consumption, small size, excellent compatibility with electronic equipment, and so on [25].

Micro-hotplate gas sensors can be divided into silicon-based [26], ceramic-based [27], glass-based [28], etc. According to the different positions of the heating electrode and the test electrode of the micro-hotplate, it can be divided into a co-planar micro-hotplate [29] and a non-planar micro-hotplate [30]. Whether the support membrane is closed can be divided into closed membrane [31] type micro-hotplate and suspended membrane [32] type micro-hotplate.

**Figure 1:**
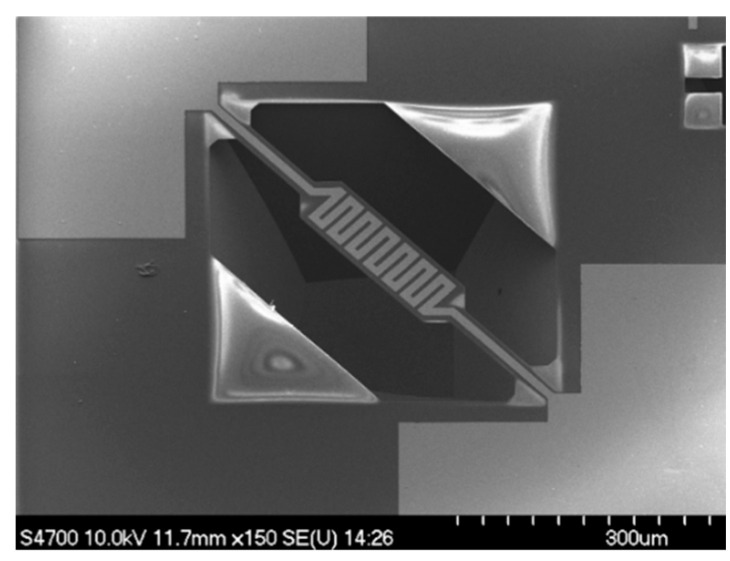
Silicon micro-hotplate [34].

Silicon-based micro-hotplates are common, and silicon-based micro-hotplates have fantastic compatibility with integrated circuits [33]. L. Xu et al. fabricated a micro-hotplate on an n-type silicon wafer, as shown in Figure 1. The thickness of the silicon wafer substrate is 350 μm. First, a silicon oxide layer is grown on the surface of the silicon wafer. Then, 1 μm low-stress SiN_0.8_ was deposited on the silicon substrate under the condition of 800 °C by using low-pressure chemical vapor deposition technology. Using the atomic layer deposition method, an aluminum oxide bonding layer is deposited, and a platinum electrode is deposited on the aluminum oxide bonding layer. Finally, the release window of the micro-hotplate is etched by an ion beam, and the micro-hotplate is released by TMAH solution [34].

During the processing of the silicon-based micro-hotplate, the heating electrode, the test electrode, the support layer, and the insulating layer materials have various choices, as shown in Table 1. The heating electrode material can be selected from Ni, Cr, Ti, Pt, Mo, etc. Test electrode materials can choose Ni, Al, Cr, Au, Ti, Pt, Pd, Ag, etc. The supporting layer and insulating layer are mainly made of SiO_2_ and Si_3_N_4_.

**Figure 2:**
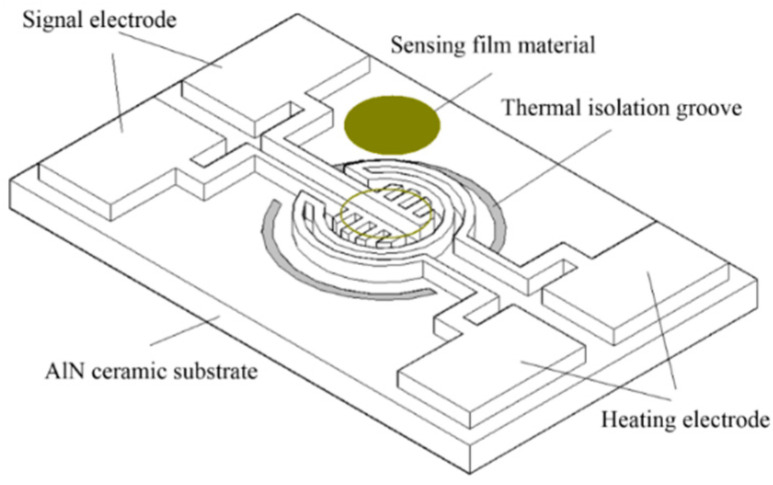
Structure of AlN ceramic micro-hotplate [42].

Compared to silicon, ceramics have a smaller coefficient of thermal expansion. Therefore, the thermal stress of the ceramic-based micro-hotplate is smaller than that of the silicon-based micro-hotplate. W. J. Zhao et al. designed a ceramic micro-hotplate based on AlN, as shown in Figure 2. A Pt test electrode and a Pt heating electrode are designed on the surface of the AlN ceramic. The heating electrode is a ring-shaped structure, the signal electrode is a comb-like structure, and the signal electrode is coated with a gas-sensitive material [42].

**Figure 3:**
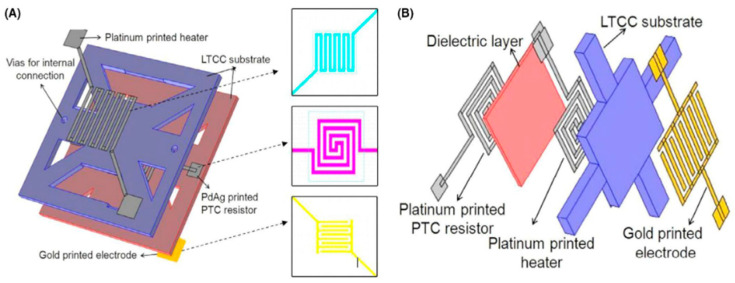
Microhotplates with different LTCC structures: (**A**) PdAg as temperature sensor; (**B**) Pt as temperature sensor [27].

Low temperature co-fired ceramic (LTCC) micro-hotplates have a wide range of applications in gas sensors and microfluidic devices. It is easy to construct three-dimensional structures. D. K. Kharbanda et al. used laser irradiation technology to sinter conductive paste on a low temperature co-fired ceramic (LTCC) substrate to generate heating electrodes and test electrodes to complete the fabrication of micro-hotplates [43]. L. Kulhari et al. designed two LTCC micro-hotplates with different structures, and the area of the micro-hotplate is 3.35 mm × 3.35 mm, as shown in Figure 3. In the first micro-hotplate structure, the PdAg temperature measuring electrode is installed in the middle of the double-layer micro-hotplate. The upper and lower surfaces are platinum heating and gold test electrodes. In the second type of micro-hotplate structure, the platinum temperature measuring electrode is installed on the bottom surface of the micro-hotplate. The middle of the double-layer micro-hotplate is a platinum heating electrode, and the surface of the micro-hotplate is a gold test electrode [27].

Compared with alumina ceramics, zirconia ceramics have extremely low thermal conductivity (2.5 W/m∙K). Compared with alumina ceramic micro-hotplates, the power consumption of micro-hotplates made of zirconia ceramics is lower. A. A. Vasiliev et al. fabricated a yttrium-stabilized zirconia ceramic micro-hotplate by sintering at a temperature of 1150 °C for 12 h using a sintering process. The film thickness is 10 μm, and the surface roughness is 0.1–0.2 μm. Platinum heater electrodes can be easily deposited on the film surface using a magnetron sputtering process. After depositing the platinum heater electrodes, the micro-hotplate was encapsulated using the TO-8 process [44]. S. Akasaka et al. fabricated a zirconia micro-hot plate oxygen sensor, and the test results showed that the sensor power consumption was greatly reduced to 80 mW, and the sensitivity to oxygen was 0.6 μA/% [45].

**Figure 4:**
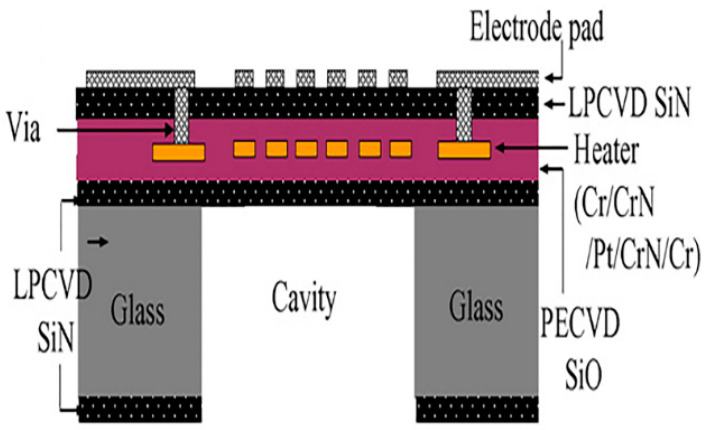
Structure diagram of glass-based micro-hotplate [46].

The thermal conductivity of glass substrates is lower than silicon substrates and ceramic substrates [25]. A typical micro-hotplate structure is shown in Figure 4. Using MEMS technology, W. Y. Chang et al. fabricated a micro-hotplate on a glass substrate, and the sensor structure is shown in Figure 4. The sensor is designed as a multilayer structure, and the heating electrode is Pt. The test results show that the power consumption is 2.35 W when the sensor is heated to 498 °C. In the stability test, the sensor can work continuously for 72 h at the heating temperature of 496.5 °C without damage [46].

Silicon-based micro-hotplates and ceramic-based micro-hotplates are mainly manufactured by MEMS technology. Hotplate has small volume and low power consumption, but its processing technology is complex [26]. Ceramic-based micro-hotplate is mainly processed by high temperature sintered ceramics, which has large volume and high power consumption, but simple processing technology.

According to whether the support layer is closed, the micro-hotplate can be divided into closed membrane and suspended membrane. Taking the silicon-based micro-hotplate as an example, the closed-film micro-hotplate has high power consumption, but the thermal plate has excellent stability. The power consumption of the suspended micro-hotplate is lower than that of the closed micro-hotplate, and the stability of the hotplate is poor. The number of suspension beams of the suspended micro-hotplate will also affect the power consumption of the micro-hotplate, as shown in Table 2. In the design process of the micro-hotplate, it is necessary to balance the relationship between the type of the support layer of the micro-hotplate, the number of cantilever beams, and the power consumption.

**Figure 5:**
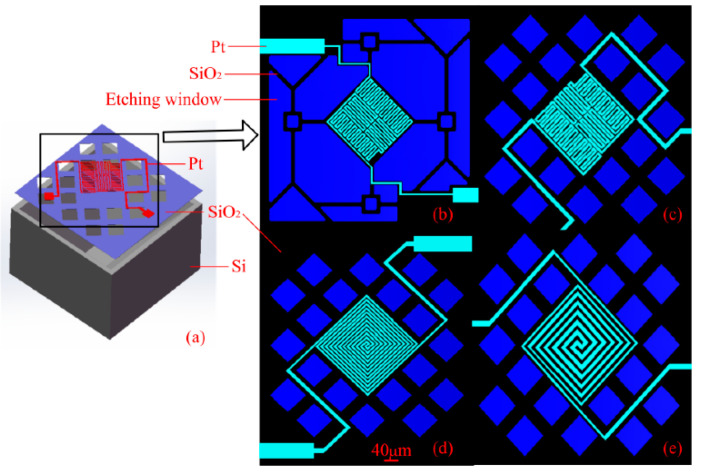
(**a**) The overall structure of the micro-hotplate, (**b**–**e**) Micro-hotplates with four different structures [32].

In addition to the conventional rectangular cantilever beam, the researchers proposed a special-shaped cantilever beam design idea to reduce the power consumption of the micro-hotplate and improve the uniformity of the temperature distribution on the surface of the micro-hotplate. Q. Liu et al. designed four kinds of special-shaped cantilever beams, as shown in Figure 5. The test results show that Design 1 has a slender cantilever beam, which can significantly reduce the power consumption of the micro-hotplate. Design 2 mesh cantilever structure has higher power consumption in Design 2 due to more shorter cantilever beams. Design 3 and Design 4 have the same cantilever beam design as Design 2, but with different heating electrodes. Design 4 has a more uniform temperature distribution on the surface of the micro-hotplate with variable width heating electrodes [32].

The temperature control methods of the micro-hotplate can be mainly divided into isothermal heating and dynamic heating. Continuous heating refers to applying a fixed voltage across the micro-hotplate to make the micro-hotplate work at a constant operating temperature. By comparing the response values of the micro-hotplate at different operating temperatures, the optimal working temperature of the micro-hotplate is determined. Q. Zhou et al. used the constant temperature test method to test the fabricated NiO-ZnO nanodisk sensor. The test results show that the sensor has a response value of 16.25 to 20 ppm SO_2_ at an operating temperature of 240 °C [51]. T.-J. Hsueh et al. used the method of isothermal heating to test the optimal operating temperature of the fabricated Au-modified Co_3_O_4_ sensor for NO_2_ response. The test results show that the sensor has the highest response value to 10 ppm NO_2_ under the working temperature of 136 °C, and the response value is 34 [52]. Q. Zhou et al. tested the synthesized SnO_2_ nanoneedles using an isothermal test method. The test results show that the SnO_2_ nanoneedles have a response value of 23.18 to 100 μL/L CO at a working temperature of 250 °C [53]. Dynamic heating refers to applying dynamic voltage at both ends of the micro-hotplate, and the voltage waveform includes rectangular wave, trapezoidal wave, triangular wave, pulse [54], etc. The dynamic response curve of the sensor is obtained by modulating the temperature of the micro-hotplate by dynamic voltage. In dynamic heating, pulse heating is typical. F. Shao et al. compared the response of ZnO nanowires to NH3 under the conditions of isothermal heating and pulse heating, and found that the response value of the sensor was higher than that of the isothermal heating condition under the condition of pulse heating. They think that the reason for the increase in the sensor response value under the condition of pulse heating is that in the low temperature range of pulse heating, there are high temperature surface oxygen species on the surface of the gas-sensing material, which enhances the sensor response. At the same time, the high temperature range has a cleaning effect on the material, which is also the reason for the enhanced sensor response [55].

### 2.2. Coat Sensitive Material of Micro-Hotplate

#### 2.2.1. Drop Coating

**Figure 6:**
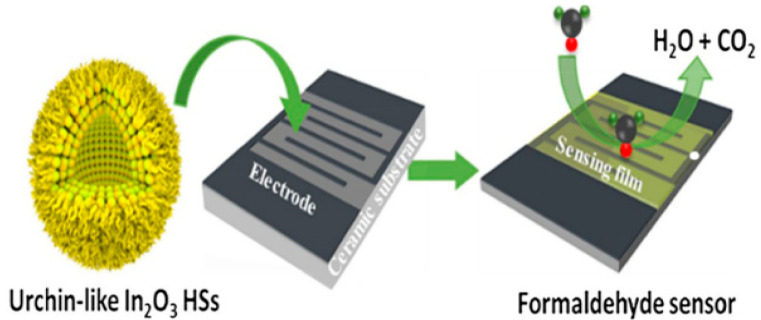
Process of sensor preparation by drop coating method [56].

By mixing the gas-sensing material with chloroplatinic acid and terpineol to form a gas-sensing slurry, a gas-sensing layer can be formed on the surface of the micro-hotplate sensor by direct drop coating or DDG droplet technology. Z. H. Tao et al. mixed the synthesized In_2_O_3_ hollow spheres with ethanol to form a slurry and coated the slurry on the ceramic micro-hotplate by a drop coating method as shown in Figure 6 [56]. W.-J. Zhao et al. mixed the synthesized In/Nb powder with chloroplatinic acid and terpineol to form a slurry, which was coated on the test electrode of the ceramic micro-hotplate. Moreover heating in an electric furnace at 500 °C for 2 h to complete the device fabrication [42]. W. Yan et al. synthesized ZnO@Co_3_O_4_ porous hollow cage, using terpineol and porous hollow cage mixed grinding, drop coating to the micro-hotplate electrode area. The micro-hotplate was heated at 250 °C for 2 h [57]. N. Luo et al. first synthesize PdRh bimetallic hollow nanocubes and mix them with ethanol to make a paste. The paste was then transferred to a Pt interdigitated electrode using a micropipette and dried at room temperature. Finally, the sensor was annealed at 150 °C for 5 h to improve sensor stability [58]. Y. Chen et al. mixed tin dioxide nanoparticles, deionized water, and triethanolamine grind using ball milling technique and drop coated the sensitive material onto a micro-hotplate using drop coating method [59]. K. Yuan et al. dispersed the prepared CeO_2_ modified WO_3_ nanowires into deionized water. Next, the dispersion liquid was dropped onto the MEMS heating sensor and dried in air at room temperature [60].

**Figure 7:**
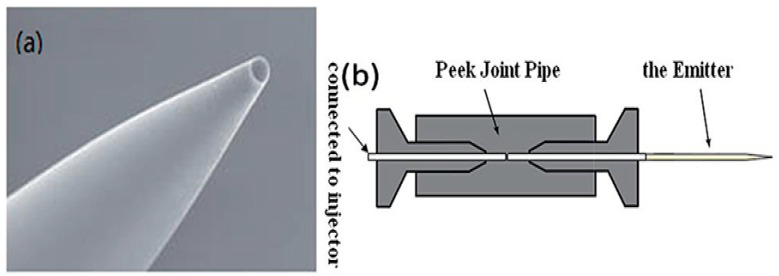
(**a**) PicoTipTM emit image. (**b**) Schematic of the emitter in combination with injector [61].

M. Li et al. developed a DDG droplet technique for small area application. First, as shown in Figure 7a, a PicoTipTM emitter having an inner diameter of 8 μm is connected to the injector through a bidirectional peek pipe, as shown in Figure 7b. The injector is filled with deionized water, driven slowly by the pump, with water dripping out of the needle tip. With the assistance of a microscope, deionized water was dropped on the micro-hotplate. Then, the sensitive material powder is sprinkled on deionized water with a spoon and left to dry. Finally, that sensor was inverted to remove redundant sensitive materials and then the sensor was placed in a muffle furnace for heating [61].

#### 2.2.2. Liquid Deposition

**Figure 8:**
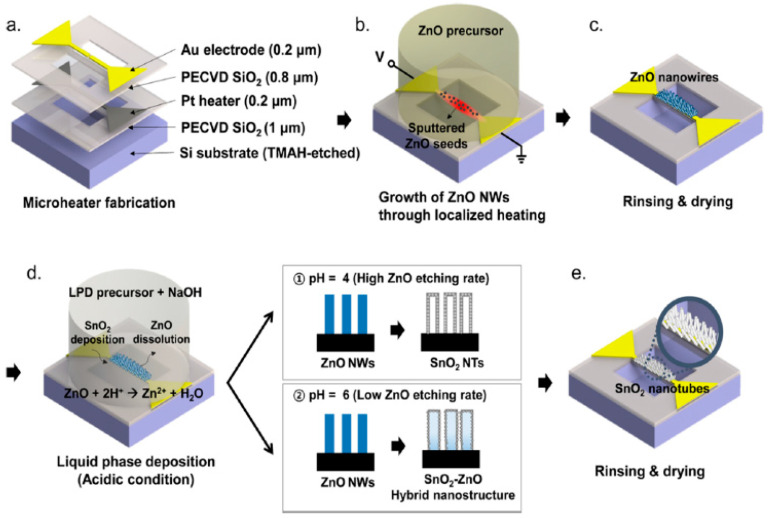
(**a**) Structural diagram of the micro-hotplate; (**b**,**c**) ZnO nanowires synthesized by hydrothermal method; (**d**,**e**) locally synthesized ZnO nanowires replaced by SnO_2_ nanotubes (PH = 4) by liquid deposition method or SnO_2_/ZnO composite structure (PH = 6) [64].

LPD is a low-temperature growth process with advantages of simplicity, easy to change film composition, and easy mass production [62]. J.-C. Chiou et al. deposited porous SnO_2_-sensing film on the sensing area of silicon-based micro-hotplate by liquid phase deposition (LPD) [63]. I. Cho et al. achieved a local liquid-phase synthesis of porous SnO_2_ nanotubes on a micro-hotplate by liquid deposition, and the process is shown in Figure 8. First, ZnO nanowires were grown in situ using a hydrothermal method. Then, a precursor solution for liquid deposition was prepared by mixing SnF_2_, HF, H_2_O_2_, H_3_BO_3_ and deionization. The PH of the precursor solution was controlled by adding NaOH. When PH = 4, ZnO nanowires were formed during a liquid deposition for 15 min. When PH = 6, a SnO_2_/ZnO composite structure was formed during liquid deposition for 15 min. Finally, the micro-hotplate was rinsed with ethanol and dried [64].

#### 2.2.3. Atomic Layer Deposition

**Figure 9:**
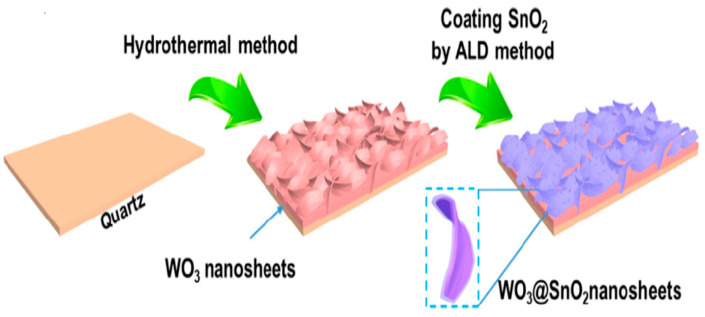
WO_3_@SnO_2_ deposition process [65].

Atomic layer deposition (ALD) has unique advantages in preparing ultrathin shell material films and precisely controlling film thickness and composition even at low temperatures. K.-P. Yuan et al. used ALD technology to prepare WO_3_@SnO_2_ composite thin films, and the response to 15 ppm NH_3_ could reach 1.55 at 200 °C. The ALD deposition process of the WO_3_@SnO_2_ composite film is shown in Figure 9. First, WO_3_ nanosheets were synthesized by the hydrothermal method. Then, SnO_2_ was deposited on the surface of WO_3_ nanosheets using an ALD deposition system (BENEQ TFS-200). The SnO_2_ film growth temperature is 150 °C, the tin source is from TDMASn, and the oxygen source is deionized water. An ALD deposition process consisted of a 0.5-s pulse of TDMASn, a 5-s pulse of argon, a 0.2-s pulse of deionized water, and a 5-s pulse of argon. WO_3_@SnO_2_ composite films with different thicknesses can be prepared by controlling the number of ALD deposition processes. Finally, the micro-hotplate with the WO_3_@SnO_2_ composite film was annealed at 550 °C for 2 h in an air atmosphere to complete the fabrication of the sensor. At an operating temperature of 200 °C, the sensor has a response value of 1.5 to 15 ppm ammonia [65].

Z. Li et al. used atomic layer deposition to deposit 20 nm ZnO films on a micro-hotplate. At the working temperature of 250 °C, the detection limit of the sensor for triethylamine reaches 22 ppb. The deposition process follows: First, diethylzinc was selected as the zinc source and water as the oxidant precursor. Diethylzinc and water were added to the atomic layer deposition vessel in sequence. The diethylzinc pulse time was 0.02 s, and the water pulse was 0.1 s. Then, a high-purity nitrogen purge was performed between successive pulses for 25 s. Finally, the ZnO thin films were annealed at 700 °C for 2 h at a heating rate of 10 °C/min in an air atmosphere. The response value of ZnO film to 10 ppm triethylamine was 7.3 under the working condition of 700 °C [66].

#### 2.2.4. Chemical Vapor Deposition

Chemical vapor deposition technology can obtain gas-sensitive thin-film materials with good crystallinity, large area and fantastic uniformity by controlling the deposition parameters [67]. Using a chemical vapor deposition method, R. L. Wilson et al. deposited NiO with different thicknesses on micro-hotplate. Under the optimal working temperature of 125 °C, NO_2_ detection is realized. The test results show that the response signal of the sensor to NO_2_ increases with the decrease of the thickness of NiO, which proves that the dependence of sensor sensitivity on Debye length applies to NiO [68].

Graphene has been used in gas sensors due to its large surface area, high sensitivity, and low electronic noise. For the large-area fabrication of graphene gas-sensing materials, chemical vapor deposition proved to be an effective method. S. Vollebregt et al. achieved wafer-level fabrication of graphene devices using chemical vapor deposition. First, a 4-inch silicon wafer was used as the substrate. Mo thin films were sputtered on top of silicon oxide using a 99.95% Mo target. Then graphene was deposited on the Mo layer using an AIXTRON BlackMagic Pro system at 1000 °C with argon, hydrogen, methane as feedstocks at a pressure of 25 mbar. After the deposition is complete, Mo is etched using phosphoric acid, and the wafer is rinsed and dried. The distance between graphene and silicon oxide is small, and the graphene will stick to the silicon oxide. Finally, Cr/Au electrical contacts are deposited using a lift-off process [69].

#### 2.2.5. Dielectrophoretic Deposition

Dielectrophoretic deposition uses a non-uniform AC electric field to generate a dipole moment to induce a dipole moment on the gas-sensitive material. The gas-sensitive material is arranged along the electric field gradient direction on the surface of the micro-hotplate. Compared with chemical vapor deposition, dielectrophoretic deposition enables room-temperature operation without the transfer process after material synthesis. The DEP deposition method is simple and has excellent repeatability and low cost [70].

**Figure 10:**
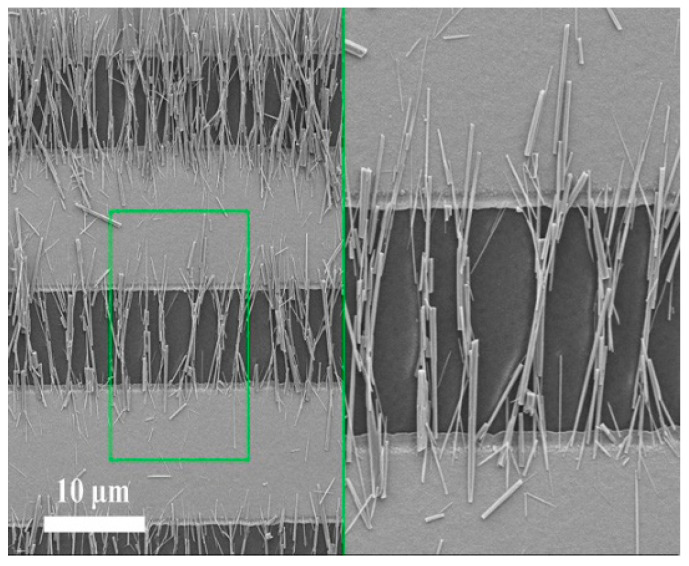
Assembly of nanowire on a micro-hotplate [55].

A dielectrophoretic deposition method, F. Shao et al. deposited ZnO nanowires on micro-hotplate. The deposition process is shown in Figure 10. First, ZnO nanowires were sonicated in isopropanol. Then the AC potential of the function generator was connected to the micro-hotplate electrode, and 5 μL ZnO nanowire solution was dripped onto the micro-hotplate by microtube. Apply a voltage of 5 MHZ, 15 V, and turn off the AC signal when the solvent evaporates. Observing the surface of the micro-hotplate by using a scanning electron microscope, the neat ZnO nanowires on the micro-hotplate surface can be observed. Finally, the sensor was annealed by heating at 400 °C for two hours [55].

**Figure 11:**
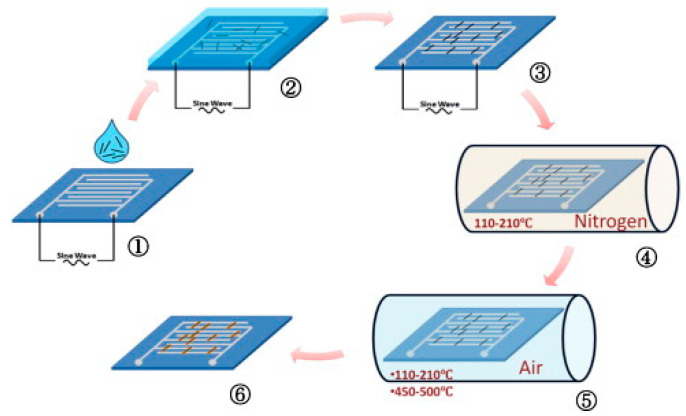
Schematic diagram of nanowire integration: ① nanowire drop-casting; ② DEP device connection; ③ nanowire row; ④ nanowire bonding; ⑤ sensor oxidation; ⑥ obtaining the final sensor [71].

X. Li et al. successfully arranged indium oxide nanowires on interdigital microelectrodes using dielectrophoresis, and the process is shown in Figure 11. First, the nanowires were added to ethanol and sonicated for 3 min. Then, transfer the nanowire suspension to a micro-hotplate using a pipette. The FG502 function generator was connected to the electrode of the micro-hotplate to generate an electric field, and a 6 V, 5 MHz sine wave was applied to realize the volatilization of ethanol. In a nitrogen atmosphere, they were heated at 110–210 °C to complete the bonding of the nanowires to the micro-hotplate. Finally, in the air atmosphere, heating at 450–500 °C completes the oxidation of the sensor [71].

#### 2.2.6. Spray Pyrolysis

**Figure 12:**
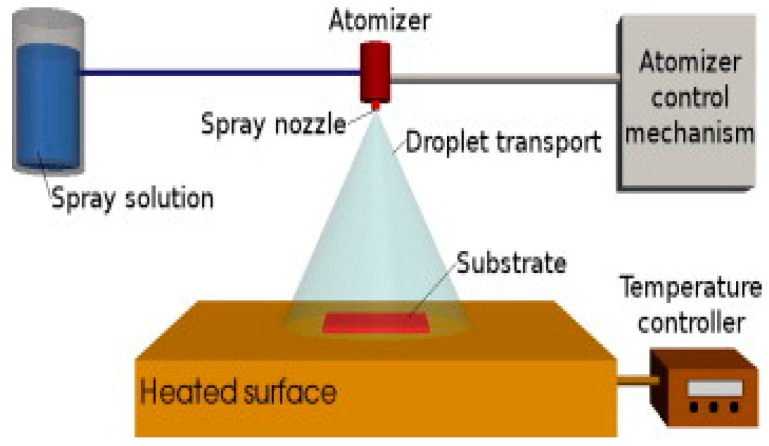
Schematic diagram of spray pyrolysis process [72].

Compared with traditional deposition, spray pyrolysis is a simple deposition method. Spray pyrolysis is mainly used for thick film, porous film, and powder production does not need vacuum conditions and is more flexible. The general scheme of spray pyrolysis deposition is shown in Figure 12, which is divided into three steps: (1) solution atomization; (2) aerosol transport; (3) precursor decomposition film growth [72].

**Figure 13:**
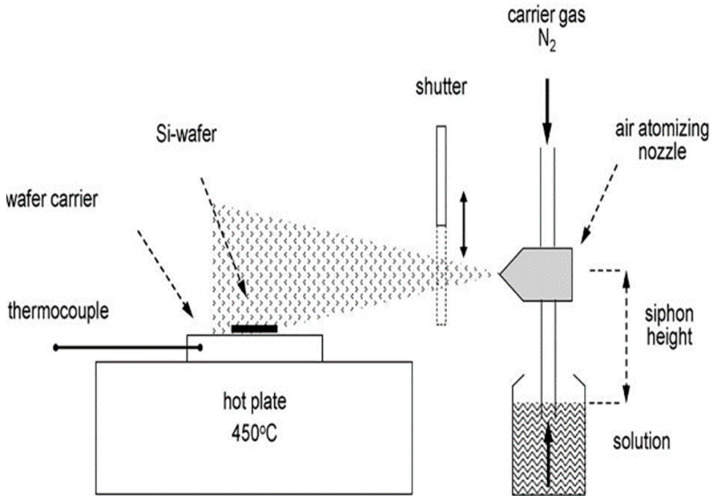
Diagram of spray pyrolysis unit [73].

Spray pyrolysis provides a simple technique for preparing thin films of various compositions. A typical spray pyrolysis apparatus is shown in Figure 13. The nozzle was placed on the right side of the hotplate at a distance of 20 cm from the sample. Silicon-base micro-hotplate simulation is carried out on a hotplate, and spray pyrolysis starts after heating for 5 min. A mechanical shutter is used between the nozzle and the micro-hotplate to control the duration of the spray [73]. However, the gas-sensitive films obtained by spray pyrolysis have poor adhesion.

#### 2.2.7. Sputtering

**Figure 14:**
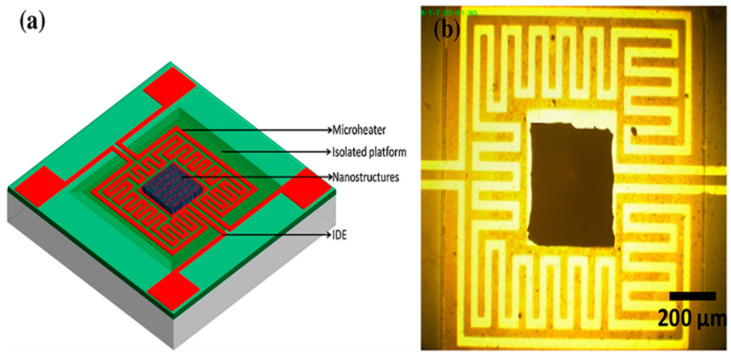
(**a**) Micro-hotplate structure, (**b**) MEMS sensor with sputtered ZnO-CuO thin film [35].

The sputter deposition of sensor-sensitive material thin films is a mature semiconductor process suitable for manufacturing mass-produced, highly repeatable gas-sensing devices. Sputter deposition allows precise control of deposition conditions, substrate temperature, and sputter power. The gas-sensitive film produced by the sputtering deposition method has the advantages of high quality and marvelous adhesion [74]. J. Zeng et al. prepared WO_3_ sensors by magnetron sputtering. The specific process is as follows: first, deionized water, acetone, and ethanol are used for cleaning the Al_2_O_3_ substrate to remove pollutants from the surface. Then, a 100 nm platinum electrode was deposited on the substrate using a radio frequency sputtering method. Finally, a tungsten film was deposited on the platinum electrode using a 2-inch round tungsten target and DPS-II ultra-high vacuum system [75]. J. G. Kang et al. deposited 120 nm Pt-modified SnO_2_ thin film on micro-hotplate by RF sputtering method and realized the detection of toluene gas [76]. L. Y. Sheng et al. prepared SnO_2_ thin films by sputtering using a Denton SJ/24LL multi-target system with a 99.99% SnO_2_ target [77]. B. Behera et al. realized the assembly of ZnO-CuO thin film and MEMS sensor by RF sputtering method, and the sensor structure is shown in Figure 14 [35].

**Figure 15:**
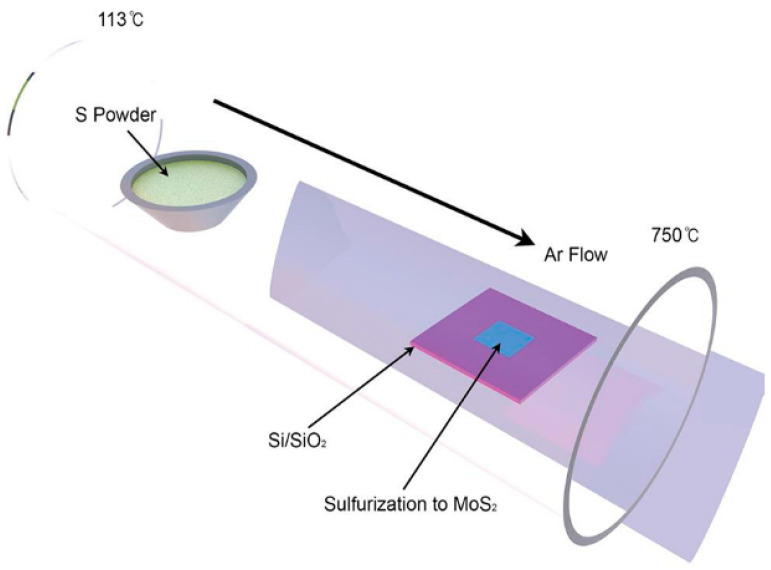
Schematic diagram of gas-phase vulcanization technology [78].

K. Lee et al. deposited a MoS_2_ thin film on a silicon substrate using a Gatan-PECS sputtering method, as shown in Figure 15. A lay of Mo is deposited on a Si substrate. Then, the Si substrate was placed in an adaptive tube furnace and heated to 750 °C at a temperature rise rate of 20 °C/min. After annealing for 30 min, the sulfur powder was heated to 113 °C and sulfur was introduced in the upstream hot zone, and the sulfur vapor completely reacted with Mo to form a MoS_2_ film [78].

#### 2.2.8. In Situ Growth

Using the vapor deposition method to deposit gas-sensitive materials on the micro-hotplate has disadvantages such as high growth temperature and expensive deposition system. Compared with vapor deposition, using the method of in situ growth on the surface of the micro-hotplate to realize the assembly of the gas-sensitive material and the micro-hotplate has the advantages of the cheap process and easy to mass produce [79].

**Figure 16:**
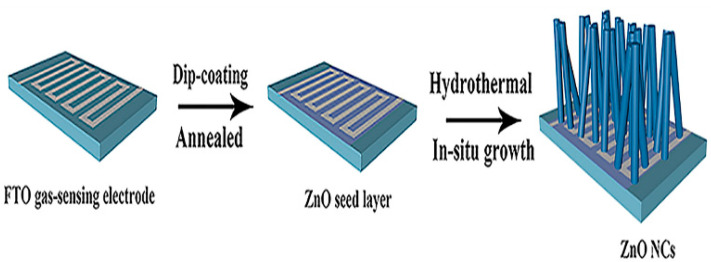
Diagram of the preparation process of in-situ growth of ZnO on FTO gas-sensing electrode [80].

J. Xuan et al. achieved the in situ growth of ZnO on the FTO gas-sensing electrode, as shown in Figure 16. Zinc acetate and diethanolamine were added to ethanol to obtain ZnO seed solution. After stirring at 30 °C for 30 min, the ZnO seed solution was added dropwise to the FTO gas-sensing electrode; the ZnO seed layer was deposited and annealed at 400 °C for 20 min. Zinc nitrate, HMTA, and PEI were added and hydrothermally treated in an autoclave at 95 °C for 6 h. Finally, the samples were rinsed with deionized water and ethanol to obtain ZnO grown in situ on the FTO gas-sensing electrode [80].

**Figure 17:**
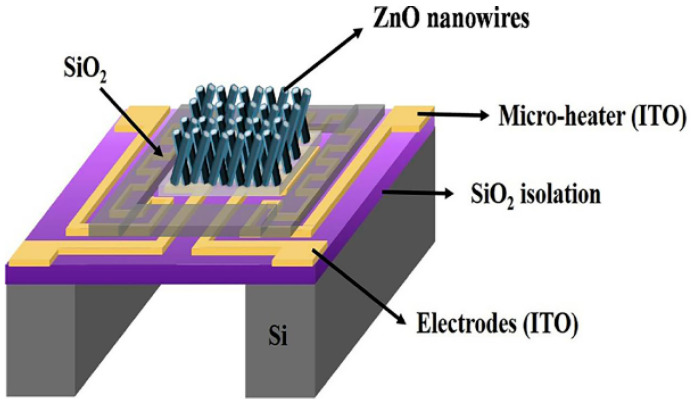
Structure of the micro-hotplate gas sensor with ZnO nanowire [82].

I. Cho et al. synthesized ZnO nanowires directly on micro-hotplate by hydrothermal reaction and realized the detection of NO_2_. The gas sensitivity of the sensor is improved by ultraviolet light activation [81]. T. J. Hsueh et al. deposited ZnO seed layer on the surface of micro-hotplate by RF sputtering method. The micro-hotplate structure for growing ZnO nanowires is shown in Figure 17 [82].

**Figure 18:**
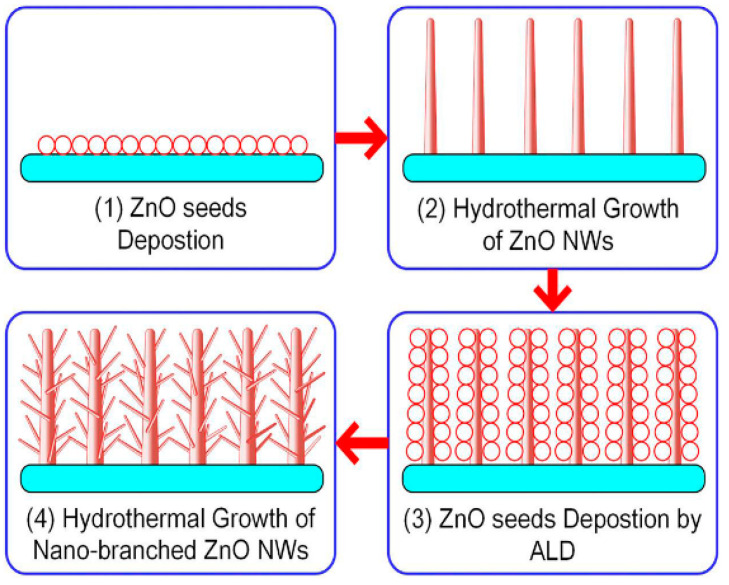
The growth process of ZnO nanowire [83].

Y. Chen et al. realized the in situ growth of ZnO nanowires on the surface of the micro-hotplate, and the specific process is shown in Figure 18. First, ZnO nanowires with diameters of 50–70 nm were synthesized in an aqueous solution. Then, with the help of a microscope, a micromanipulator is used to drop zinc acetate ethanol solution on the sensing area of the micro-hotplate. They covered a zinc acetate seed film on the surface, heating to 350 °C in the air for 20 min to generate a nano zinc oxide seed layer. Finally, ZnO nanowires are grown on the surface of the WAF in situ by adopting a hydrothermal method [83].

#### 2.2.9. Inkjet Printing

**Figure 19:**
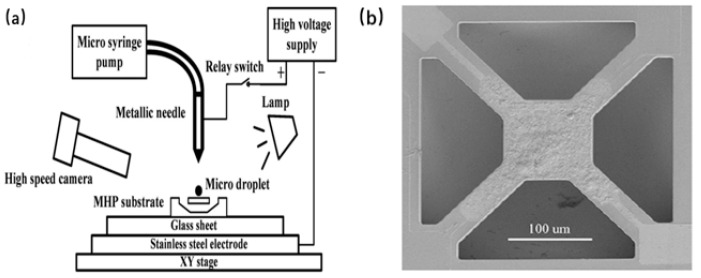
(**a**) EHD inkjet printing system. (**b**) Finished EHD printed micro hotplate surface [85].

The assembly of gas-sensitive material and micro-hotplate can be realized using inkjet printing technology. EHD inkjet printing is a printing technology capable of producing droplets more minor than the inner diameter of the nozzle. But inkjet printing technology requires expensive, sophisticated equipment [84]. H. Wu et al. achieved the assembly of Pd-modified SnO_2_ nanofibers on a micro-hotplate using EHD inkjet printing. The EHD inkjet printing device is shown in Figure 19a, including metal needles, micro-injection pumps, relays, high-speed cameras, high-voltage power supplies, light sources, glass plates, and stainless steel electrodes. During the EHD inkjet printing process, the microsyringe pump replenished the Pd-modified SnO_2_ nanofiber ink into the metal needle at a rate of 2 μL/min. The stainless steel electrode is ground, the relay is turned on, and the metal needle receives a high voltage of 2 KV. The spherical ink droplet first hangs on the top of the metal needle. When the relay is disconnected, the high-voltage electric field generates electrostatic force, pulling the ink droplet into a cone shape. The ink droplet is separated from the top of the Taylor cone and falls on the surface of the micro-hotplate. Repeat the above steps until the ink completely covers the micro-hotplate test electrode area, as shown in Figure 19b [85].

**Figure 20:**
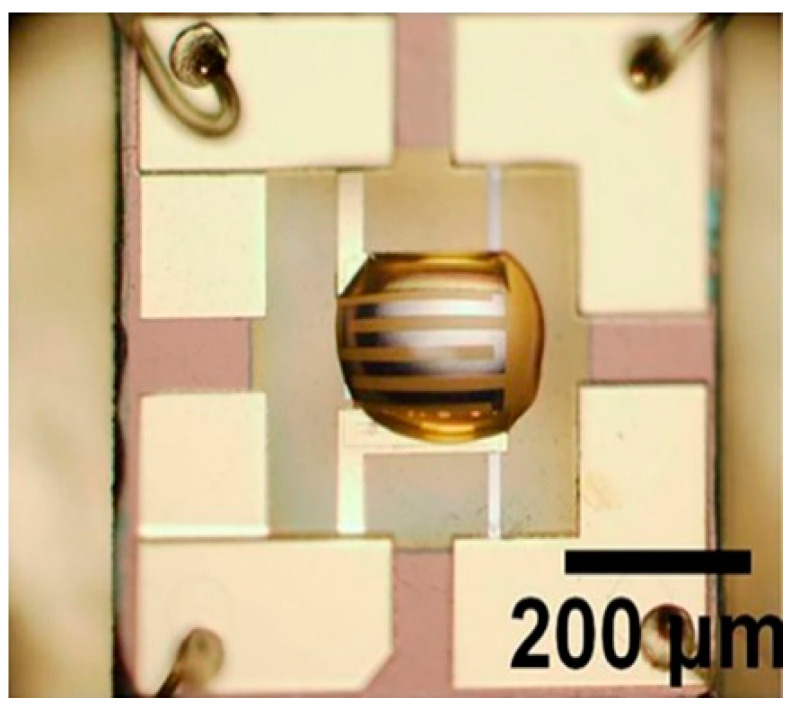
Inkjet printing of an optical image of SnO_2_ nanoparticle ink deposited on a micro-hotplate [86].

M. A. Audio et al. added glycerol to SnO_2_ particles and then subjected the mixture to ultrasonic treatment to prepare the ink required for printing. SnO_2_ nanoparticle ink was printed onto a micro-hotplate using a JetLab II inkjet print system. After printing, the micro-hotplate was dried at 300 °C for 3 h. The sensor surface obtained after inkjet printing is shown in Figure 20 [86].

#### 2.2.10. Screen Printing

**Figure 21:**
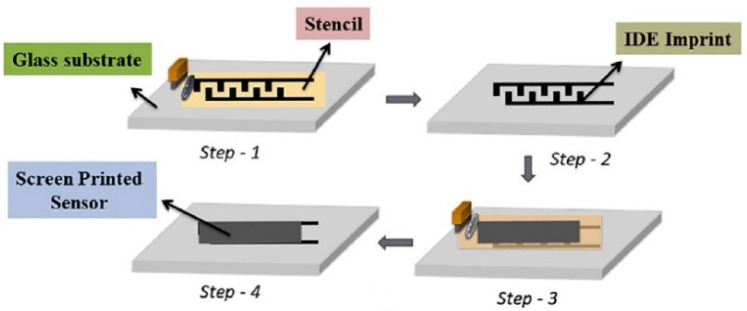
Screen-printing process [87].

The sensitive material and the sensor array can be directly assembled using the screen printing method for the sensor array with a large area. Using screen-printing process, A. Ibrahim et al. fabricated a Pd/g-C_3_N_4_ gas sensor for hydrogen detection. The screen-printing process is shown in Figure 21. First, the interdigitated electrode pattern was printed on a glass substrate and then heated to 120 °C for curing. Then, 20 mg of Pd/g-C_3_N_4_ material was added to terpineol to make a screen-printing paste. To ensure the effect of screen printing, a 135-micron screen was chosen with a 2-inch rubber squeegee to spread the gas-sensitive material evenly on the electrode area of the micro-hotplate. Finally, the sensor was annealed for 30 min [87].

**Figure 22:**
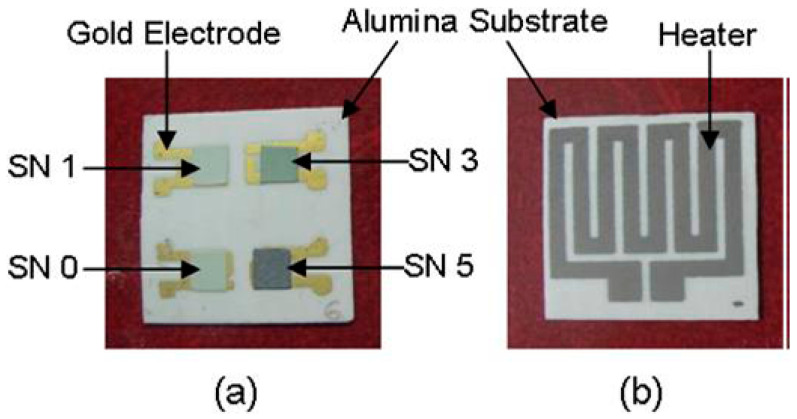
Sensor array, (**a**) front, (**b**) back [88].

M. Choudhary et al. printed tin oxide slurry doped with copper oxide on the surface of the test electrode of the sensor by screen-printing technology to form a sensor array and realize the detection of H_2_S, as shown in Figure 22. The sensor array consists of four sensors, the front side of the sensor array is a test electrode, and the backside is a heating electrode [88]. W. Lu et al. used a high-resolution screen printer (MT650) to print a platinum-decorated alumina gas-sensitive film on the surface of a quartz-based micro-hotplate to realize the detection of methane [89].

#### 2.2.11. Self-Assembly

**Figure 23:**
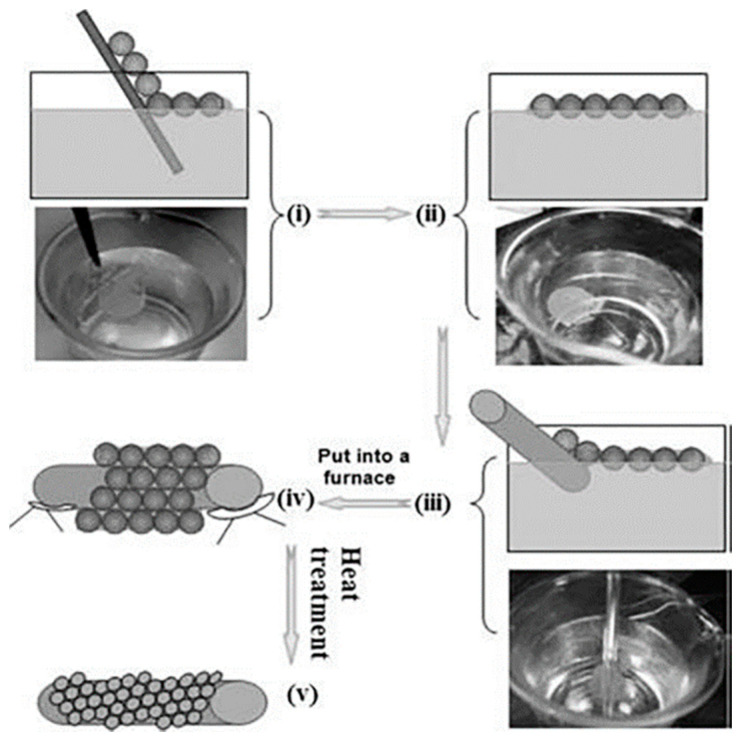
(**i**) Glass plate covered with PS colloid immersed in the solution. (**ii**) Monolayer of colloid floating on the surface of the solution. (**iii**) Pick up with glass rod. (**iv**) Drying. (**v**) Calcination to remove the PS microspheres [90].

The self-assembly technique proposed by Decher to fabricate ultrathin films by electrostatic interaction. The most significant difference between the ultrathin films prepared by self-assembly technology and those prepared by vapor deposition and spin coating is that the films prepared by self-assembly technology are highly ordered. F. Q. Sun et al. have realized the assembly of SnO_2_ thin films on curved surfaces by using the method of self-assembly. The self-assembly process is shown in Figure 23. First, the glass plate covered with PS colloid was immersed in the precursor solution, and the monolayer film would float on the surface of the solution, and the film was picked up by the curved glass rod. The glass rod covered with the film is then dried. Finally, the PS microspheres were removed by calcination. This method allows porous ordered thin films to form on any curved surface [90].

**Figure 24:**
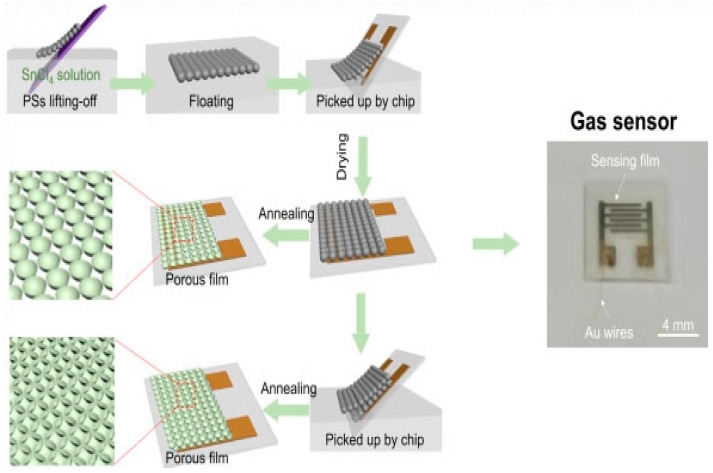
PS microspheres preparation of SnO2 porous membrane [91].

Y. Xu et al. used polystyrene microspheres to prepare SnO_2_ porous films, as shown in Figure 24. First, the single-layer polystyrene microspheres are put into the SnCl_4_ solution. Due to surface tension, polystyrene microspheres were suspended on the surface of SnCl_4_. Then, an interdigital electrode was used for fishing out the polystyrene microspheres. Finally, the polystyrene microspheres were removed by heating the interdigital electrode to 400 °C in air and calcining for 2 h. Both double-layer and triple-layer SnO_2_ films can be manufactured using polystyrene microsphere templates [91].

**Figure 25:**
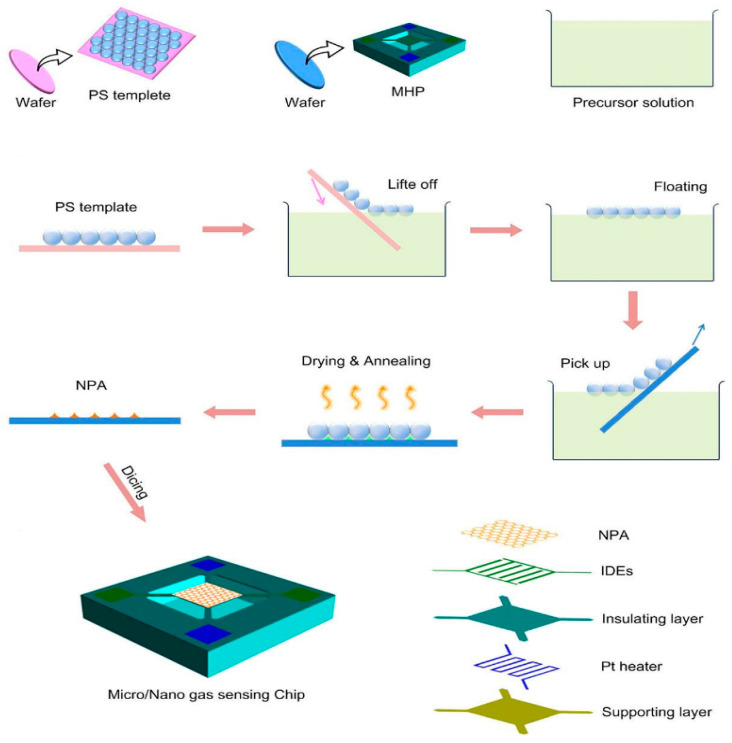
Sensor wafer-level array film formation [49].

The self-assembly method can assemble sensitive materials and sensor array for the gas sensor array with a small area and large quantity. Using the in situ assembly method, L. Xu et al. made the nanopore array film on the wafer-level micro-hotplate collection. They achieved the goal of producing thousands of gas-sensing units simultaneously, as shown in Figure 25. The PS microsphere template is floated in the precursor solution. The sensitive material, the microsphere template, and the micro-hotplate are assembled by fishing wafers covered with the micro-hotplate units. By annealing at 400 °C for 2 h, the microsphere template was removed, and an ordered porous gas-sensitive film was formed on the wafer surface. After the wafer-level assembly of the gas-sensitive thin film is realized, the wafer is cut according to the number of units required by the sensor array [49].

### 2.3. Comparison of Coating Methods for Gas-Sensitive Materials

Among the many coating methods for gas-sensitive materials, the drop coating method is easy to operate and has a high gas response value, but the coating thickness is difficult to control. Liquid deposition is a low-temperature growth process that does not require complex equipment and the substrate is not limited. The thin film prepared by liquid deposition method has a dense structure, but the sensor response is low. Atomic layer deposition (ALD) is a method of depositing material layer by layer on the surface of a substrate in the form of a single atomic film. Atomic layer deposition can control the film thickness by controlling the number of reaction cycles, but atomic layer deposition (ALD) film deposition is slow and inefficient. Chemical vapor deposition technology can obtain gas-sensitive thin-film materials with marvelous crystallinity, large area, and fantastic uniformity by controlling the deposition parameters. Dielectrophoretic deposition can operate at room temperature without the transfer process after material synthesis, and the deposition method has low cost and excellent repeatability. However, it is difficult to set the voltage amplitude, frequency, and period of DEP deposition.

Spray pyrolysis is mainly used for the assembly of thick film materials and does not require vacuum conditions. The film raw materials are mixed in a solution state and are not prone to agglomeration. However, the films formed by spray pyrolysis have poor adhesion. Sputtering deposition is a mature semiconductor process. By controlling deposition conditions, substrate temperature, deposition power, and other conditions, high-quality, high-adhesion films can be prepared. However, sputtering deposition of thin films requires higher deposition conditions, generally requiring vacuum conditions.

In situ growth is accomplished by arranging a seed layer on the surface of the micro-hotplate, and using the induction method to complete the growth of the seed layer. The films prepared by in situ growth have excellent adhesion, but the preparation process is complicated.

The inkjet printing technology uses the ink prepared from the gas-sensitive material to form a Taylor cone at the nozzle using electrostatic force, producing material droplets smaller than the nozzle, and realizes the assembly of the gas-sensitive material. The inkjet printing technology is easy to operate, but the formed film material is a thick film type, the film thickness is difficult to control, and the inkjet printing equipment is expensive. Screen printing technology uses screen and rubber squeegee to spread the gas-sensitive material evenly on the surface of the micro-hotplate in a large area. The film-forming method is simple and efficient. However, the film thickness is difficult to control. The films prepared by the self-assembly method are highly ordered, easy to operate, and have fast sensor response. Table 3 shows the comparison of coating methods for gas-sensitive materials.

## 3. Conclusions

In this paper, we introduce the classification standard and structure of micro-hotplates and summarize the assembly process and characteristics of different gas-sensitive materials and micro-hotplates, such as: drop coating, liquid deposition, atomic layer deposition, chemical vapor deposition, electrophoretic deposition, spray pyrolysis, sputtering, in situ growth, inkjet printing, screen printing, self-assembly, etc.

The effective formation of the patterned nano-gas-sensitive material layer at a specific position on the surface of the micro-hotplate gas sensor is the leading technical obstacle to be overcome in the manufacturing of the micro-hotplate gas sensor. Gas-sensitive material film with high stability, excellent adhesion, and marvelous uniformity is prepared on the surface of the micro-hotplate to realize the reliable assembly of the gas-sensitive material and the micro-hotplate, which can improve the stability and response value of the micro-hotplate gas sensor.

Compared with other coating methods, the in situ growth and self-assembly method can form a porous gas-sensitive material film with marvelous adhesion, does not need expensive equipment, has the advantages of the cheap process, easy to expand, and so on. Therefore, it can be predicted that in situ growth and self-assembly will play an essential role in developing gas-sensitive materials and micro-hotplate assembly technology.

## Figures and Tables

**Table 1:** Silicon-based micro-hotplates material parameters.

Support Layer	Heating Electrode	Insulation	Test Electrode	Reference
SiO_2_	Ni	SiO_2_	Ni	[35]
SiO_2_	Ni	SiO_2_	Al	[36]
SiO_2_	Cr/Pt	SiO_2_	Cr/Au	[37]
SiO_2_	Ti/Pt	SiO_2_	Ti/Pt	[38]
SiO_2_	Pt	SiO_2_/Si_3_N_4_	Pt	[39]
SiO_2_	Ni	SiO_2_	Pd/Ag	[40]
SiO_2_/Si_3_N_4_	Mo	Si_3_N_4_	-	[41]

**Table 2:** Micro-hotplate power consumption parameters.

Membrane Type	Platform	Number of Cantilevers	Power (mW)	Temperature	Reference
Closed	Si/SiO_2_	-	100	259	[35]
Closed	Si_3_N_4_/SiO_2_	-	100	260	[47]
Closed	Si/SiO_2_	-	280	250	[36]
Suspended	Si/SiO_2_	1	2.96	400	[48]
Suspended	Si/SiO_2_	2	18	400	[34]
Suspended	Si/SiO_2_	4	30	350	[49]
Suspended	Si/SiC	6	35	380	[50]

**Table 3:** Comparison of thin film coating methods.

Coating Method	Operating Temperature	Professional Setting	Film Features
Drop Coating	Normal	No	The method is simple and the sensor response value is high, but the film thickness is difficult to control.
Liquid Deposition	Low	No	The thin film is dense and the sensor response is low.
Atomic Layer Deposition	Low	Yes	The film uniformity is excellent, but the film deposition rate is slow.
Chemical Vapor Deposition	High	Yes	The film has marvelous crystallinity and large area, but the deposition temperature is high, and impurities are easily introduced during the deposition process.
Dielectrophoretic Deposition	Normal	No	The thin film deposition method is simple and has excellent repeatability, but it is difficult to set the deposition voltage amplitude, frequency, and period.
Spray Pyrolysis	High	Yes	The film is a thick film material, and the film raw material is not easy to agglomerate, but the film adhesion is poor.
Sputtering	Low	Yes	Film adhesion is high, however, vacuum conditions are required for film deposition.
In Situ Growth	Low	No	The film has excellent adhesion, but the preparation process is complicated.
Inkjet Printing	Normal	Yes	The film material is thick film type, the film thickness is difficult to control, and the equipment price is high.
Screen Printing	Normal	Yes	The film formation efficiency is high, but the film thickness is difficult to control.
Self-assembly	Normal	No	The film is highly ordered and has a fast response time

## Data Availability

Not applicable.
